# Growth of High Material Quality Group III-Antimonide Semiconductor Nanowires by a Naturally Cooling Process

**DOI:** 10.1186/s11671-016-1443-4

**Published:** 2016-04-26

**Authors:** Kan Li, Wei Pan, Jingyun Wang, Huayong Pan, Shaoyun Huang, Yingjie Xing, H. Q. Xu

**Affiliations:** Key Laboratory for the Physics and Chemistry of Nanodevices and Department of Electronics, Peking University, Beijing, 100871 China; Division of Solid State Physics, Lund University, Box 118, S-22100 Lund, Sweden

**Keywords:** VLS mechanism, Chemical vapor deposition, Naturally cooling growth

## Abstract

We report on a simple but powerful approach to grow high material quality InSb and GaSb nanowires in a commonly used tube furnace setup. The approach employs a process of stable heating at a high temperature and then cooling down naturally to room temperature with the nanowire growth occurred effectively during the naturally cooling step. As-grown nanowires are analyzed using a scanning electron microscope and a transmission electron microscope equipped with an energy-dispersive X-ray spectroscopy setup. It is shown that the grown nanowires are several micrometers in lengths and are zincblende InSb and GaSb crystals. The FET devices are also fabricated with the grown nanowires and investigated. It is shown that the grown nanowires show good, desired electrical properties and should have potential applications in the future nanoelectronics and infrared optoelectronics.

## Background

Among various III–V semiconductors, group III-antimonides, e.g., InSb and GaSb, show various attractive properties, such as high carrier mobilities, narrow band gaps, small carrier effective masses, and large g factors. These properties make the group III-antimonide nanowires the ideal material systems for applications in high-speed electronics, infrared optoelectronics, and quantum devices [1–7]. In particular, InSb nanowires have played an essential role in the recent experimental search for Majorana fermions in the solid state—an elusive class of elementary Fermion states whose anti-fermion states are themselves [8, 9]. As the continuous progresses in both the device applications and the fundamental research, growth of high material quality group III-antimonide nanowires is still on highly demanding.

Sophisticated techniques have been exploited to grow high-quality group III-antimonide nanowires, such as metal-organic vapor phase epitaxy (MOVPE), molecular beam epitaxy (MBE), and chemical beam epitaxy (CBE) [1, 10–14]. However, due to the high running cost and delicate growth conditions, the massive growth of long group III-antimonide nanowires in a reasonably low cost has been difficult with these expensive techniques [15, 16]. Chemical vapor deposition (CVD) is a high-yield and inexpensive technique to prepare nanowires, including narrow band gap InSb and GaSb nanowires [17, 18]. In CVD experiments, vapor-liquid-solid (VLS) mechanism [19] is usually employed to synthesize III–V semiconductor nanowires. Growth temperature, catalyst metal, and source supply are considered as the main control factors in the growth of nanowires by the VLS mechanism [1]. For example, InSb and GaSb nanowires have been prepared on substrates coated with Au catalyst via the VLS mechanism in an environment containing enough precursors at stable heating temperatures [1, 20]. Ambipolar behavior is a typical characteristic of a field effect transistor (FET) made with a narrow band gap semiconductor. However, such an ambipolar performance has been rarely observed in FETs made from these CVD-grown nanowires [1, 20], which cast a severe doubt in using these nanowires for device applications. Another severe problem in the CVD growth of group III-antimonide nanowires is the yield or successful rate. A sensitive dependence of InSb nanowire growth on the substrate temperature has been reported [17]. Detailed studies have revealed that the temperature window for the growth of InSb nanowires is very small (less than 20 °C) and can appear in an entirely different temperature region at different growth experiments with different settings in other growth parameters [17, 18]. This has posed a strict limitation on the CVD growth of group III-antimonide nanowires with a good successful rate.

Here, we present a convenient method with a high successful rate for the growth of InSb and GaSb nanowires in a commonly used tube furnace setup. The method employs a process of heating at a given high temperature for a certain time and then letting the system be cooled down naturally, and the nanowires are grown in the slow, naturally cooling step. As-grown nanowires are analyzed using a scanning electron microscope (SEM) and a transmission electron microscope (TEM) equipped with an energy-dispersive X-ray spectroscopy (EDX) setup. It is seen that a high successful rate of almost 100 % is achieved in the growth of InSb and GaSb nanowires by this approach, and the grown nanowires are several micrometers in lengths and are high material quality, zincblende InSb and GaSb crystals. The FET devices are also fabricated with the grown nanowires and characterized by electrical measurements. It is shown that the grown nanowires show good, desired electrical properties.

## Methods

**Fig. 1:**
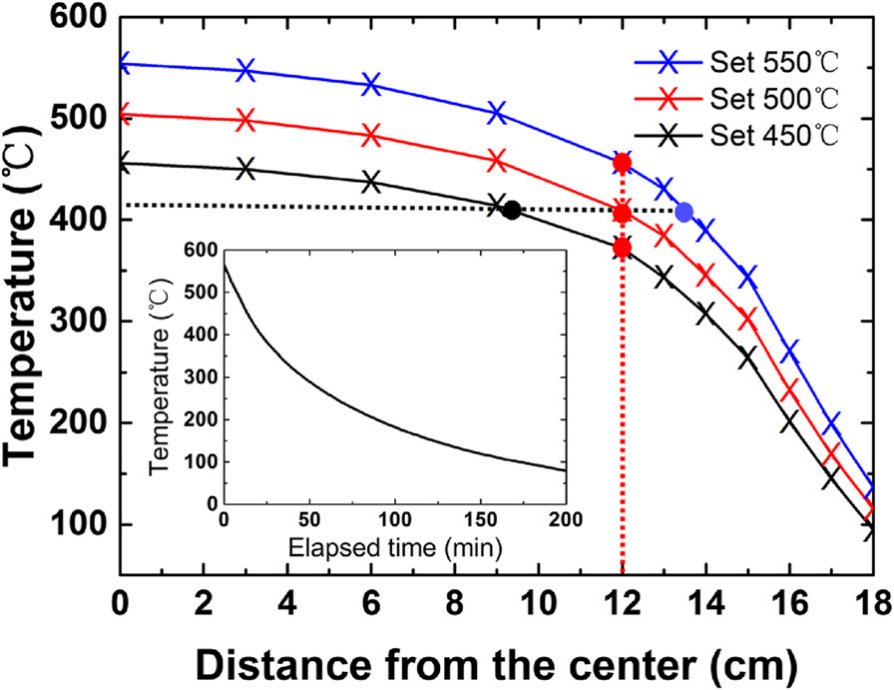
Temperature distributions along the tube furnace axis with tube center set at different heating temperatures. The *inset* shows a representative curve for the decrease in temperature at the furnace center as a function of time during natural cooling after a stable heating at a temperature of 580 °C

In our nanowire growth experiments, a single-temperature-zone tube furnace (Lindberg TF55035C) is used. The furnace is first characterized with the measurements of the temperature distributions along the furnace axis under different heating temperatures. Figure 1 shows the measured temperature distributions along the furnace tube axis at heating temperatures 550, 500, and 450 °C. It is shown that the temperature is a non-linear function of the position in the furnace. But it is generally seen that for a given heating temperature at the center of the furnace, the temperature is lower at a remoter position from the center. There are two possible ways to obtain a desired growth substrate temperature in a tube furnace. A most commonly used one is to set a stable heating temperature in the center of the furnace and adjust the substrate position to obtain a desired temperature for nanowire growth using a temperature distribution guideline as shown in Fig. 1. Another one is the method we are proposing here, namely, to place a growth substrate at a properly chosen position in the furnace and to approach the desired growth temperature by varying temperature at the heating position, as indicated in principles by the red dashed line across different temperature curves in Fig. 1. A naturally cooling process after the furnace has been heated up to a preset high temperature is a good example for such a varying furnace temperature process, as is shown in the inset of Fig. 1 where a representative process of temperature decrease, from a heating temperature of 580 °C, at the center of the furnace as a function of elapsed time during the naturally cooling period is shown. Our proposed growth method does not require a precise control of the substrate position in a growth furnace and of heating temperature and can be conveniently used to grow those nanowires with a very small growth parameter space. Below, we will first demonstrate successful applications of our method to grow InSb nanowires, the materials with an extremely small growth temperature window.

## Results and discussion

**Fig. 2:**
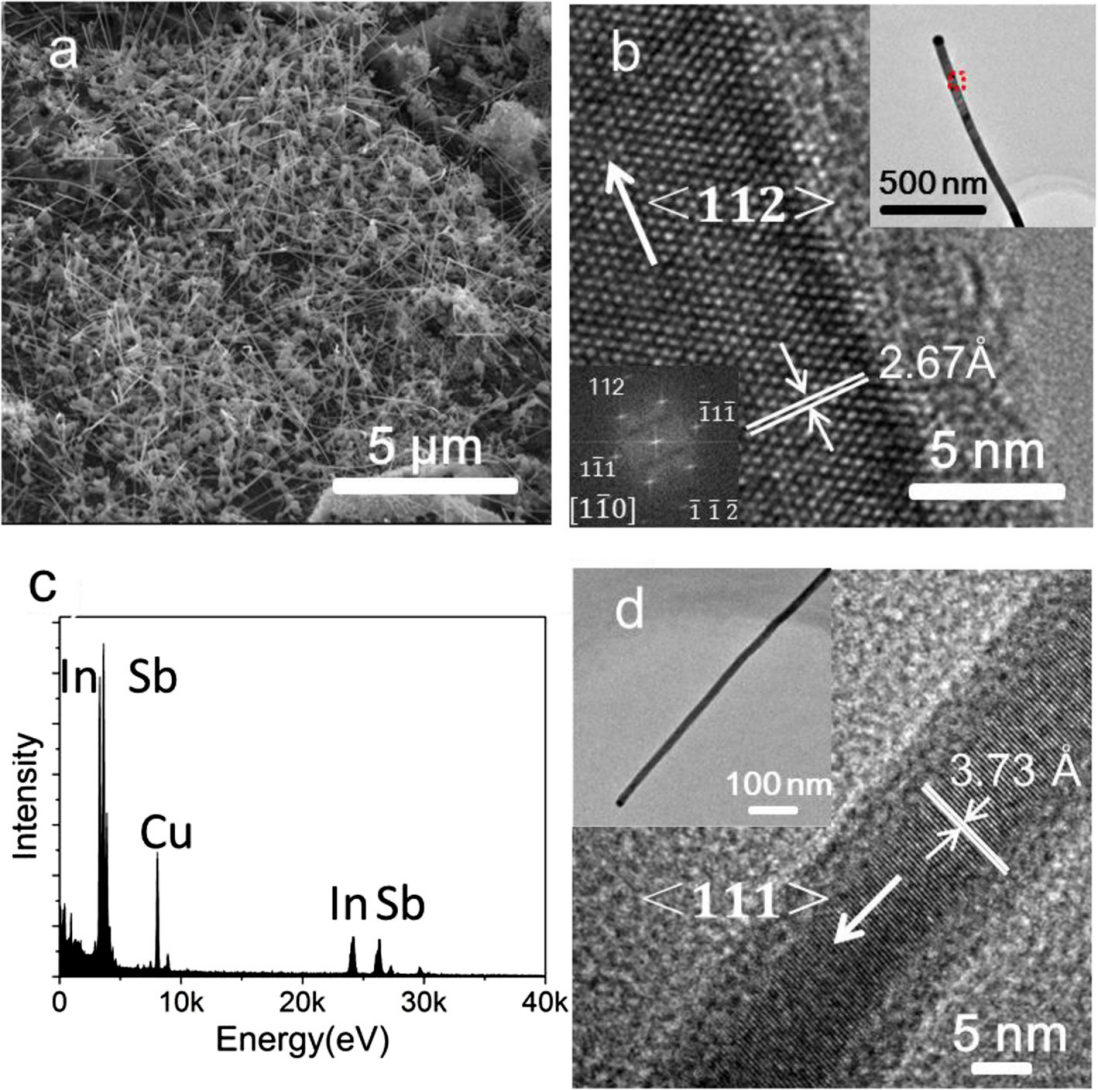
**a** SEM image (45° tilted angle view) of InSb nanowires grown with an InSb powder source placed at the tube furnace center and an Au nanoparticle-coated Si/SiO_2_ substrate at 12 cm away from the source by heating the source at 550 °C for 1 h and then letting the system cool down naturally to room temperature. **b** HRTEM image of a segment of an as-grown InSb nanowire. The diameter of the nanowire is ~25 nm and the nanowire has a zincblende crystal structure. The *upper right inset* shows a low-magnification TEM image of the nanowire. The *red square* in the *inset* indicates the segment of the nanowire with the HRTEM image shown in the main panel. The *lower left inset* shows the FFT pattern corresponding to the HRTEM image shown in the main panel. **c** EDX spectrum of the nanowire. The ratio of In:Sb in the nanowire is close to 1:1. **d** HRTEM image of a 10-nm-diameter nanowire grown using the same approach as above except for setting the heating at a temperature of 580 °C for 10 min. The *inset* is a low-magnification TEM image of the nanowire

In growth for InSb nanowires, a piece of Si wafer covered with a SiO_2_ layer (300 nm in thickness) is used as a substrate. Droplets of water solution containing 10-nm-diameter gold particles are deposited on the cleaned Si/SiO_2_ substrate and the substrate is then transferred to an incubator to evaporate the solvent. Commercially available InSb powder (Sichuan Xinlong, purity 99.999 %) is used as a source and is placed on a quartz boat located at the center of a quartz tube in the furnace. The growth substrate is placed at a downstream position, about 12 cm from the source. The system is then evacuated using a rotary pump for several hours and flushed with argon for several times before heating. A wide range of furnace heating temperatures (450–580 °C) is tested for the growth of InSb nanowires. The temperature of the substrate is about 80 °C lower than a furnace heating temperature. The stable heating time is about an hour in this experiment and then the system is cooled down to room temperature naturally (approximately in 3 h). The pressure is about 150 Torr during the heating period. Hydrogen is used as a carrier gas in both the stable heating and the naturally cooling period. Figure 2a shows an SEM image of as-grown InSb nanowires obtained by heating the quartz tube at temperature 550 °C for 1 h and then cooling down the system naturally. Here, it is seen that dense nanowires are grown on the substrate. Most of the nanowires have lengths of more than 5 μm and diameters of 20–80 nm. Figure 2b shows a TEM image of an as-grown nanowire with a diameter of 25 nm (upper right inset), a high-resolution TEM (HRTEM) image of a section of the nanowire as indicated by a red square in the upper right inset (main panel), and its corresponding FFT pattern (lower left inset). Here, in the upper right inset, a round particle at the tip of the nanowire is clearly seen, which indicates the nanowire is most likely grown by the VLS mechanism. It is also seen that the nanowire is a single zincblende crystal without visible extended defects and dislocations and is oriented along a <112> direction with an inter-planar spacing of 2.67 Å, consistent with the value of bulk InSb crystal (2.65 Å). An EDX spectrum of the nanowire is presented in Fig. 2c, which shows that the nanowire consists of 51 at.% In and ~49 at.% Sb, suggesting a good stoichiometric composition. Here, we note that such HRTEM and EDX analyses have been carried out for more than 20 InSb nanowires grown in this process and all these nanowires are found to show the same structural properties.

The results presented above show that good-crystal-quality InSb nanowires can be grown in a large scale in a quartz tube in a process of heating and natural cooling. For a given quartz tube and a setup for source and substrate positions, heating temperature and heating period are the two main tuning parameters in the growth of InSb nanowires. Although the parameter space for the growth of long InSb nanowires using a sophisticate technique such as MOVPE or CBE is normally very small and is hard to control, the heating temperature and heating period can be tuned in a wide range for the growth of InSb nanowires with lengths in several micrometers in a simple quartz tube setup using our method. Figure 2d shows a TEM image (inset) and a HRTEM image (main panel) of an InSb nanowire grown in our method by setting the heating at a temperature of 580 °C for 10 min. It is seen that the grown InSb nanowire has a diameter of ~10 nm and is grown along a <111> crystallographic direction. No extended defects and dislocations are found in this nanowire either. We note that this thin nanowire is grown along a different crystallographic direction from the thicker nanowire shown in Fig. 2a, b. The dependence of the growth direction on the diameter of semiconductor nanowires has been reported and discussed in the literature [21, 22]. The reason for the formation of thin nanowires here is due to the fact that a much shorter heating time used in this growth experiment has prevented particle aggregation and has thus helped maintaining the original size of catalyst Au nanoparticles for the growth of thin nanowires [23, 24].

**Fig. 3:**
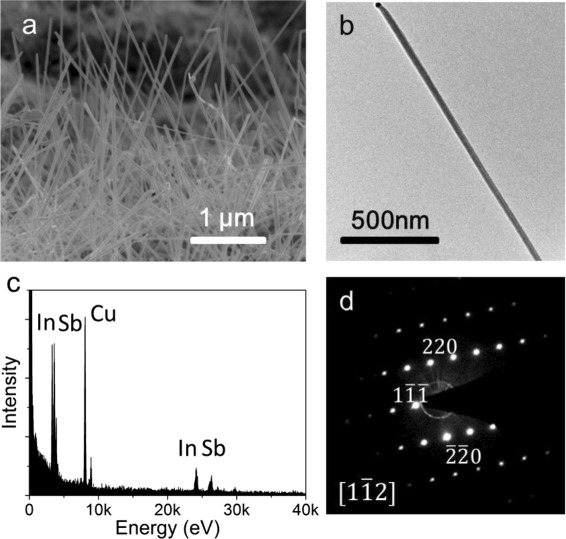
**a** SEM image (80° tilted angle view) of InSb nanowires grown with an individual In particle source placed at the center of the furnace and an Sb particle source at an upstream position. The substrate is placed at a downstream position, 12 cm away from the source. The heating at the furnace center is set at 550 °C for 1 h and then the system is cooled down naturally to room temperature. **b** HRTEM image of an as-grown InSb nanowire. **c** EDX spectrum of the nanowire, where the ratio of In:Sb close to 1:1 can be extracted. **d** FTT pattern for a selected segment of the nanowire, which indicates that the nanowire is a zincblende crystal

Growth of InSb nanowires can also be achieved in the same heating and naturally cooling process using separated In and Sb sources. Figure 3a shows an SEM image of the InSb nanowires grown by using In and Sb particles as sources in a quartz tube. Here, in the experiment, the In particle is placed in the center of the furnace and the Sb particle is placed in an upstream position. The stable heating is carried out at a temperature of 550 °C for 1 h. It is found that the nanowires are several micrometers in length and about 25 nm in diameter. Figure 2b shows a TEM image of a single InSb nanowire grown in this way. The corresponding EDX spectrum is shown in Fig. 3c, where a good stoichiometric ratio (52.5 at.% In and 47.5 at.% Sb) is observed. The FFT pattern obtained for a selected segment of the nanowire is shown in Fig. 3d, and it is seen again that the nanowire is a zincblende crystal. To our best knowledge, this is the first report on the growth of InSb nanowires by using separated In and Sb sources in a one-temperature-zone furnace system.

**Fig. 4:**
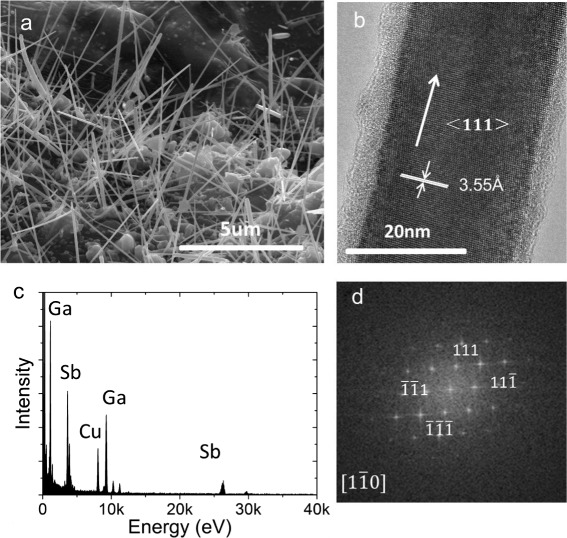
**a** SEM image (45° tilted angle view) of GaSb nanowires grown with a GaSb powder source placed at the furnace center and a growth substrate at a downstream position, 12 cm away from the source. The heating is set at a temperature of 600 °C for 1 h and then the system is cooled down naturally to room temperature. **b** HRTEM image of an as-grown GaSb nanowire with a diameter of ~25 nm. **c** EDX spectrum of the nanowire, where the ratio of In:Sb close 1:1 can be extracted. **d** FFT pattern obtained from the HRTEM image shown in **b**

Our heating and natural cooling method is applicable to the growth of other semiconductor nanowires. As an example, we show in Fig. 4 GaSb nanowires grown by the method using GaSb powder (Sichuan Xinlong, purity 99.999 %) as the source. We have tested a wide range (570–670 °C) of heating temperatures in the growth of GaSb nanowires. The substrate has been placed at a position to obtain a substrate temperature higher than 500 °C during the heating period. Figure 4a shows an SEM image of the GaSb nanowires grown by setting the heating at a temperature of 600 °C for an hour and the substrate temperature at ~500 °C during the heating. The grown GaSb nanowires are seen to have lengths of more than 5 μm and diameters of 20–80 nm. Figure 4b shows a HRTEM image of an as-grown GaSb nanowire, where the nanowire is seen to grow along a <111> direction without observable extended defects and dislocations. The inter-planar spacing (3.55 Å) is close to that of bulk zincblende GaSb crystal (3.52 Å). The EDX analysis shown in Fig. 4c reveals a good stoichiometric composition of ~48.5 at.% Ga and ~51.5 at.% Sb in the nanowire. The FFT pattern of the HRTEM image is shown in Fig. 4d, confirming the single zincblende crystalline structure of the GaSb nanowire. All these results demonstrate the effectiveness of our heating and naturally cooling technique for the nanowire growth.

### Device Fabrication and Measurements

To demonstrate that the grown nanowires show good desired electrical properties, FET devices are fabricated from as-grown InSb nanowires on a Si/SiO_2_ substrate as described in [25, 26] and are characterized. In the device fabrication, first, InSb nanowires are mechanically transferred from the growth substrate to a Si/SiO_2_ substrate, on which alignment marks and contact pads are predefined. After resist spinning, source and drain window regions with a spacing of about 500 nm are then exposed and opened on individual nanowires by electron beam lithography (EBL) technique. The defined areas are then etched in (NH_4_)_2_S_x_ solution to remove native oxide layers on the nanowires. Subsequently, Ti/Au (5/90 nm in thickness) metal electrodes are fabricated by metal deposition via electron beam evaporation (EBE) and lift-off process. Next, after a second resist spinning step, the channel regions of the InSb nanowires are exposed and opened by a second EBL process for gate-insulator-layer deposition. A 10-nm-thick high-*k* HfO_2_ dielectric thin film is fabricated on the channel regions by atomic layer deposition at 90 °C and lift-off process. A third resist spinning and EBL step is applied to open gate electrode windows, which overlap slightly with the source and drain electrodes to cover the entire channel regions. Then, 5/90-nm Ti/Au metal electrodes are prepared by EBE and lift-off process. The final FET devices are wire-bonded and characterized by electrical measurements in a physical property measurement system.

**Fig. 5:**
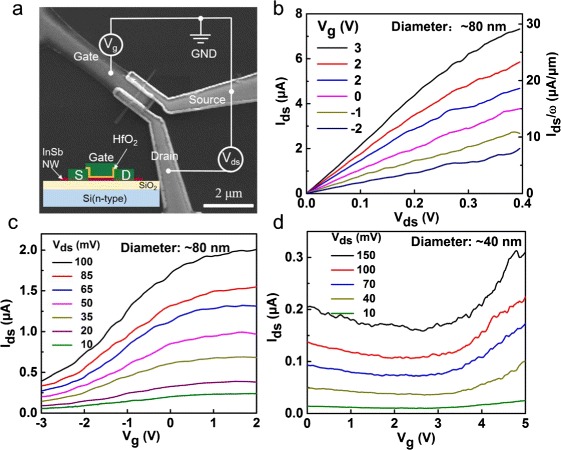
**a** SEM image of a FET device made from an InSb nanowire grown in this work. The *inset* is a schematic view of the device. **b** Output characteristics of an 80-nm-diameter InSb nanowire FET device measured at different gate voltages at room temperature. **c** Transfer characteristics of the nanowire FET device measured at different source-drain voltages at room temperature. **d** Transfer characteristics of a 40-nm-diameter InSb nanowire FET device measured at different source-drain voltages at room temperature

Figure 5a shows an SEM image of a fabricated nanowire FET made from an 80-nm-diameter InSb nanowire grown using our heating and naturally cooling technique and the corresponding measurement circuit setup. The cross-sectional structure along the nanowire axis of the device is illustrated schematically in the inset. Figure 5b shows the output characteristics of the FET device at room temperature under gate voltage *V*_*g*_ from −2 to 3 V. The linear *I*_ds_–*V*_ds_ curves seen in the low-voltage region indicate good ohmic contacts in the device at room temperature. Clearly, a large on-state current *I*_on_ as high as 7.3 μA is observed at *V*_ds_ = 400 mV and *V*_*g*_ = 3 V, giving rise to a normalized current of *I*_on_*/L* = ~29 μA/μm. Here, *L* is the circumference of the nanowire. The large on-state current could benefit from the single crystalline nature of the InSb nanowire. Figure 5c shows the transfer characteristics of the device at room temperature under source-drain voltage *V*_ds_ ranging from 10 to 100 mV. The extracted electron field effect mobility is 69 cm^2^/*Vs* at *V*_ds_ = 100 mV. Further improvement in the mobility is possible when reducing surface adsorption on the nanowire and device substrate [27, 28]. There is no obvious ambipolar behavior seen in Fig. 5c, partly because the large diameter (80 nm) of the nanowire degrades the gate efficiency. The transfer characteristics of a 40-nm-diameter InSb nanowire FET device are presented in Fig. 5d. Here, obvious ambipolar behaviors are observed. This is a direct evidence for a FET made from a narrow band gap semiconductor. The large off-state current (~0.16 μA at *V*_ds_ = 150 mV) reflects the fact that the thermal excitation limits the minimum off-state current in the narrow band gap semiconductor at room temperature [28]. Above measured characteristics confirm the intrinsic properties of InSb nanowires.

## Conclusions

In summary, we have demonstrated a simple approach to grow high material quality InSb and GaSb nanowires in a tube furnace setup. We show that growth of the nanowires with several micrometers in length can be achieved during natural cooling after a stable heating at a high temperature. FET devices are fabricated from the grown nanowires, and the electrical measurements show that these grown nanowires exhibit desired electrical properties. Our work demonstrates that the heating and naturally cooling process we have developed for nanowire growth is a general approach and could be used to grow many other semiconductor nanowires. The approach is particularly powerful for the growth of those nanowires with a very small growth temperature window.

## Abbreviations

CVD: chemical vapor deposition
EBL: electron beam lithography
EDX: energy-dispersive X-ray
FET: field effect transistor
FFT: fast Fourier transformation
SEM: scanning electron microscope
TEM: transmission electron microscope
VLS: vapor-liquid-solid
